# A90 ASSESSING 30-DAY MORTALITY IN PATIENTS UNDERGOING EMERGENT ADD-ON ENDOSCOPIC GI PROCEDURES

**DOI:** 10.1093/jcag/gwad061.090

**Published:** 2024-02-14

**Authors:** K Healey, M Borgaonkar, J McGrath

**Affiliations:** Memorial University of Newfoundland Faculty of Medicine, St. John's, , Canada; Department of Medicine, Memorial University of Newfoundland, St. John's, , Canada; Department of Medicine, Memorial University of Newfoundland, St. John's, , Canada

## Abstract

**Background:**

Gastrointestinal (GI) endoscopic procedures may need to be performed emergently on hospitalized patients. Current short-term mortality rates following emergent GI endoscopic procedures are attributed to their indication and vary by geographic area, ranging from 1.89% to as high as 10%. Recent studies describing clinical outcomes and mortality of non-scheduled GI endoscopic procedures are limited.

**Aims:**

This study aimed to describe the 30-day mortality and clinical outcomes of patients who received endoscopic intervention as an add on, non-scheduled procedure over a 2-month period across 2 tertiary hospitals, in St. John’s, Newfoundland and Labrador.

**Methods:**

We retrospectively reviewed the charts for all patients between July 1, 2021, and August 31, 2021, who received an esophagogastroduodenoscopy (EGD), colonoscopy, sigmoidoscopy, and percutaneous gastrostomy (PEG) across 2 tertiary hospitals in St. John’s, Newfoundland and Labrador. Endoscopic procedures on hospitalized patients that were added to the emergency endoscopy list were included. Procedures were done during working hours on weekdays or outside working hours at night-time, and during the weekend. Data on demographics, endoscopic findings, treatment outcomes, and 30-day mortality, were collected through electronic chart review. Descriptive statistics were conducted for data analysis.

**Results:**

Of the 258 add on procedures performed on 213 patients, 17 patients (8.0%) died within 30 days of having had the procedure. The most frequently performed procedure was EGD, and the mortality associated with it was 9.4% (12/127), followed by sigmoidoscopy (1/29, 3.4%), colonoscopy (0/18, 0%), PEG (3/9; 33.3%). One patient who died had both EGD and colonoscopy (1/25, 4%). No patients died from both EGD and sigmoidoscopy (0/5, 0%). The median time between the procedure and death was 15 days. The median age was 72 years old. Most patients received endoscopic intervention for suspected bleeding. The major causes of death were cardiac, GI, malignancy, respiratory, and other.

**Conclusions:**

Hospitalized patients are a distinct cohort with competing risks that need to be considered when planning endoscopic procedures. Further investigation is necessary with a larger patient cohort over multiple years.

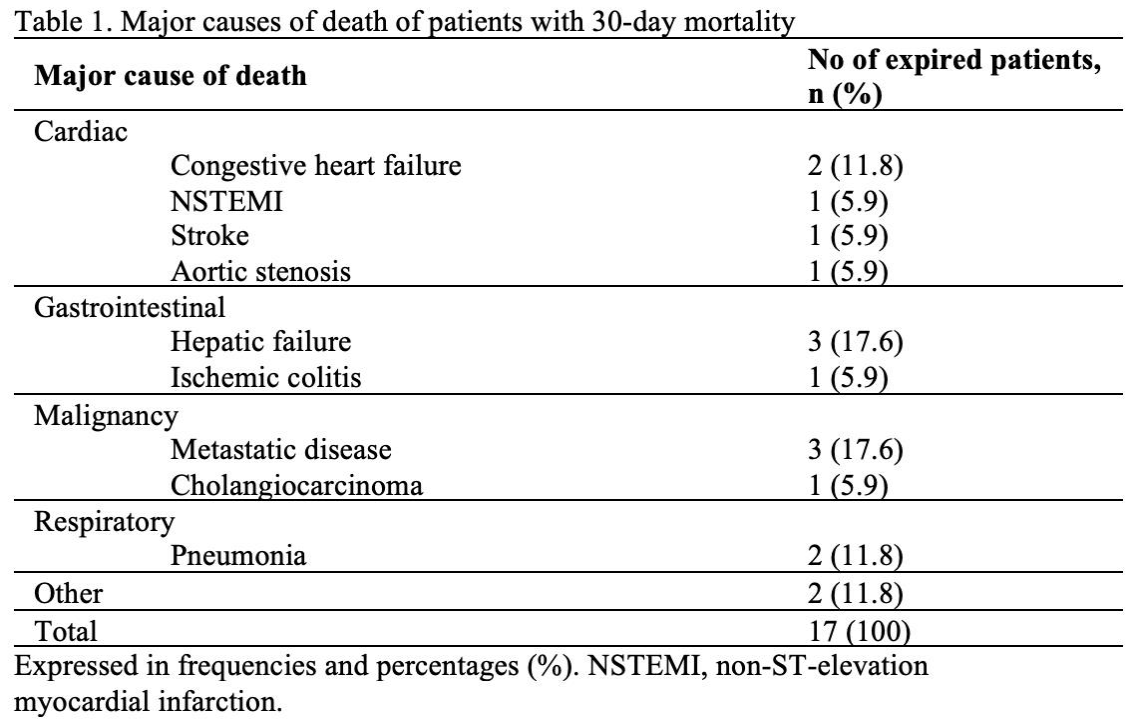

**Funding Agencies:**

Summer Undergraduate Research Award (Memorial University of Newfoundland)

